# Supplementary material for: Human neuronal excitation/inhibition balance explains and predicts neurostimulation induced learning benefits
Source: PLoS Biol. 2023 Aug 31;21(8):e3002193. doi: 10.1371/journal.pbio.3002193 (PMC10470965; doi:10.1371/journal.pbio.3002193)
Supplement: Attachment — Submitted filename: Response to Reviewers.pdf [file pbio.3002193.s002.pdf]

Tracking Number [PBIOLOGY-D-22-02703R1]

Dear Editorial team from Plos Biology,

We would like to thank you for your recent response to our submission of the manuscript entitled 'Explaining and Predicting the Effects of Neurostimulation via Neuronal Excitation/Inhibition on Learning'. We were pleased to hear from the previous handling editor (Kris Dickson) and current editor (Lucas Smith) that we were allowed to submit a revised version if we addressed the points raised by the reviewers. We appreciate the time and effort that went into the reviewing process, and we were delighted to see that the reviewers recognize the merits and strengths of our study, while also providing constructive comments. We feel that we have addressed all the comments and we describe below how this has been done on a point-by-point basis. Revisions that were made in the manuscript are non-indented and blue in the rebuttal letter and marked as track changes in the revised manuscript.

Kind regards,

Roi Cohen Kadosh & Nienke van Bueren in the name of the authors

Reviewer #1:

We would like to thank the reviewer for the important comments, mainly about the lack of theoretical validity and the questions around the quality of the spectral model fits. We have now addressed these comments in the following ways:

1) Lack of theoretical validity: The study is based on a link of the aperiodic exponent to E/I-balance (Gao2017). This link has been established in LFP and ECoG data, quote: "we propose that slope changes in a particular frequency region (30-70 Hz) correspond to changes in E:I balance, while making no claims about other frequency regions", as also referenced by Fig 1D in the current article. In the study here, the exponent was estimated on the frequency range of 1-40 Hz for one 1 frontal EEG electrode (with frontal electrodes also prone to artifacts). So in terms of relating exponent to E/I as measured here, this link is not justified. Without a justified relationship of the used aperiodic exponent measure to excitation balance, it is not possible make inferences about the presumably excitatory mechanism of tRNS solely based on this aperiodic marker.

We would like to thank the reviewer for this notion of the possible lack of theoretical validity regarding the link between the aperiodic exponent and the E/I balance. We have discussed this important issue with other researchers in the field, including Bradley Voytek, to gain a better understanding as many recent studies have linked the aperiodic exponent to E/I-balance (e.g., Cellier et al. (2021), McSweeney et al. (2023), Dave et al., 2018, Donoghue 2020a & 2020b), He et al. (2019), Hill et al. (2022), McSweeney et al. (2021), Ostlund et al. (2022)).

Fortunately, a recent study mitigates the concern that the reviewer expressed. Namely, our theoretical framework gains further validity from the recent eLife paper by Chini et al. (2022). They used computational modelling, animal data, optogenetics, and human EEG in the 5–20 & 5–45 Hz range, which are comparable to our frequency range. Chini et al.'s results converge on a similar result to Gao et al. (2017). Thus, showing that the exponent can provide a valid measure of the E/I balance. For more clarity, we have added this recent paper in our manuscript on line 85 and revised Figure 1D accordingly. We also included the following part in the Methods of the manuscript on line 584: 'We would like to point out that the study by Gao et al. (2017) found this link in the 30-70 Hz range using local field potential (LFP) and electrocorticography (ECoG) data, while making no claims about other frequency ranges such as the ~1-40 Hz range that is frequently studied in cognition (i.e., Cellier et al., 2021; He et al., 2019; Mcsweeney et al., 2023). However, a more recent study from Chini et al. (2012) that used computational modelling, animal data, optogenetics, and more importantly human EEG data in the 5-20 Hz/5-45 Hz range, showed a similar result as Gao et al. (2017). Thus, showing that the exponent can provide a valid measure of the E/I balance.'

- Chini, M., Pfeffer, T., & Hanganu-Opatz, I. (2022). An increase of inhibition drives the developmental decorrelation of neural activity. *ELife*, *11*, e78811. https://doi.org/10.7554/eLife.78811

We acknowledge that the frontal electrodes are prone to muscle artefacts. However, we removed this typical noise with Independent Component Analysis (ICA) and manually double-checked for the presence of artefacts. In addition, since the rs-EEG measurements were eight minutes long, we made sure that we included enough good quality EEG data in the analyses for extracting the exponent. We have included this on line 571 of the manuscript: 'The exponent was calculated for the midline frontal electrode Fz (which is the electrode we focused on in our preregistration), which is the closest to the stimulation electrodes F3 and F4. This is motivated by previous studies that have shown that Fz has been repeatedly involved in processes that are related to mathematical learning or other types of learning (Holm et al., 2009; Morís et al., 2013). We acknowledge that the frontal electrodes are prone to muscle artefacts. However, we removed this typical noise with Independent Component Analysis (ICA) and manually double-checked for the presence of artefacts.'

2) Missing quality control of the chosen model fits: The procedure of estimating the aperiodic exponent involves fitting a model on power spectra. For this work, the quality of the spectral model fit cannot be assessed because 1) crucial model parameter settings are not reported in the methods (e.g. how many peaks were fit, was a knee parameter, was Welch's method used to estimate the spectra, etc.), 2) and more importantly: there is no report of model fit quality, making it not possible to assess whether reported differences are simply due to differences in model fit quality. There are no visualizations of the data spectra, making it not possible to assess data quality. Also raw exponent values are not reported, only the exponent change. Given the wide spread of possible exponent changes visible in Fig. 3 it would be great to get more insight into this.

1) We thank the reviewer for the remark regarding the missing model parameter setting in our manuscript. We have added this in the Methods of the manuscript on line 577. 'The following FOOOFGroup settings were used: peak_width_limits=[1, 8], max_n_peaks=5, with no knee fitted to the data.' Please note that our script to extract the aperiodic activity is also openly available. Additionally, we have added a referral to the Welch's method on line 564: 'The rs-EEG data of the remaining participants were separated in 2-second segments with an overlap of 1 second and windowed with a Hann window, using the Welch's method.'
2) To report model fit quality, we have added two plots in the Supplementary Information. One concerning the raw exponent values for the pre and the post EEG measurement. We have also noted the goodness of fit and the error of the fit in the figure caption. We have adjusted this accordingly in the manuscript and refer to these plots on line 579.
   '**Figure S6** shows the raw exponent values together with the goodness of fit and the error of the fit for both the baseline values (R^2=.96, error=.11) and the post measurement values (R^2=.96, error=.11). Additionally, we have plotted the averaged power spectra for the four different conditions at baseline as shown in **Figure S7**.'

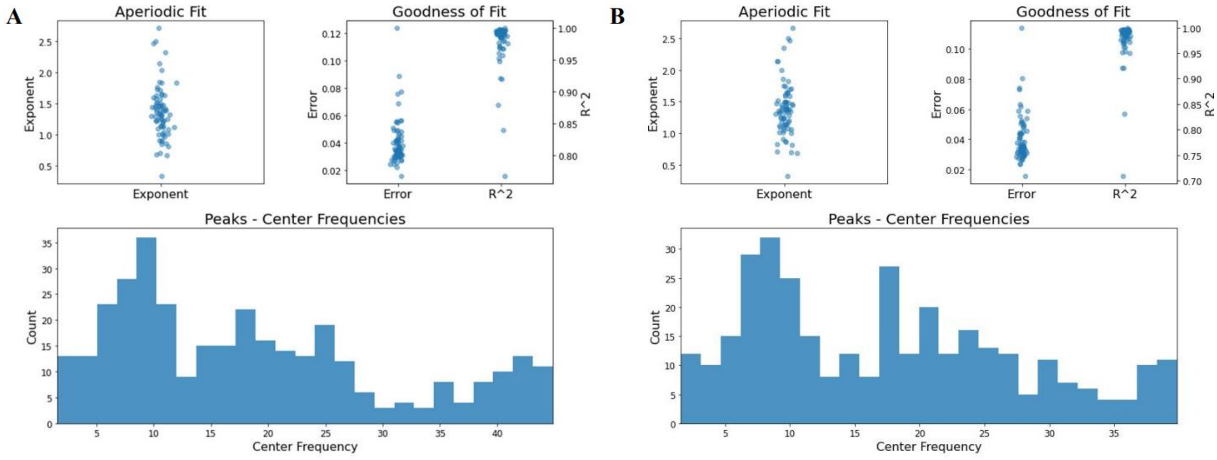

Figure S6| Raw exponent values for the baseline and post measurement. A) Individual baseline exponent values are indicated in the top left panel. The top right panel indicates the error of the fit and the R^2. This plot shows a small error (mean=.04) and a high goodness of fit (mean R^2=.97). **B)** Individual post measurement exponent values are indicated in the top left panel. The top right panel indicates the error of the fit and the R^2. This plot shows a small error (mean=.11) and a high goodness of fit (mean R^2=.96).

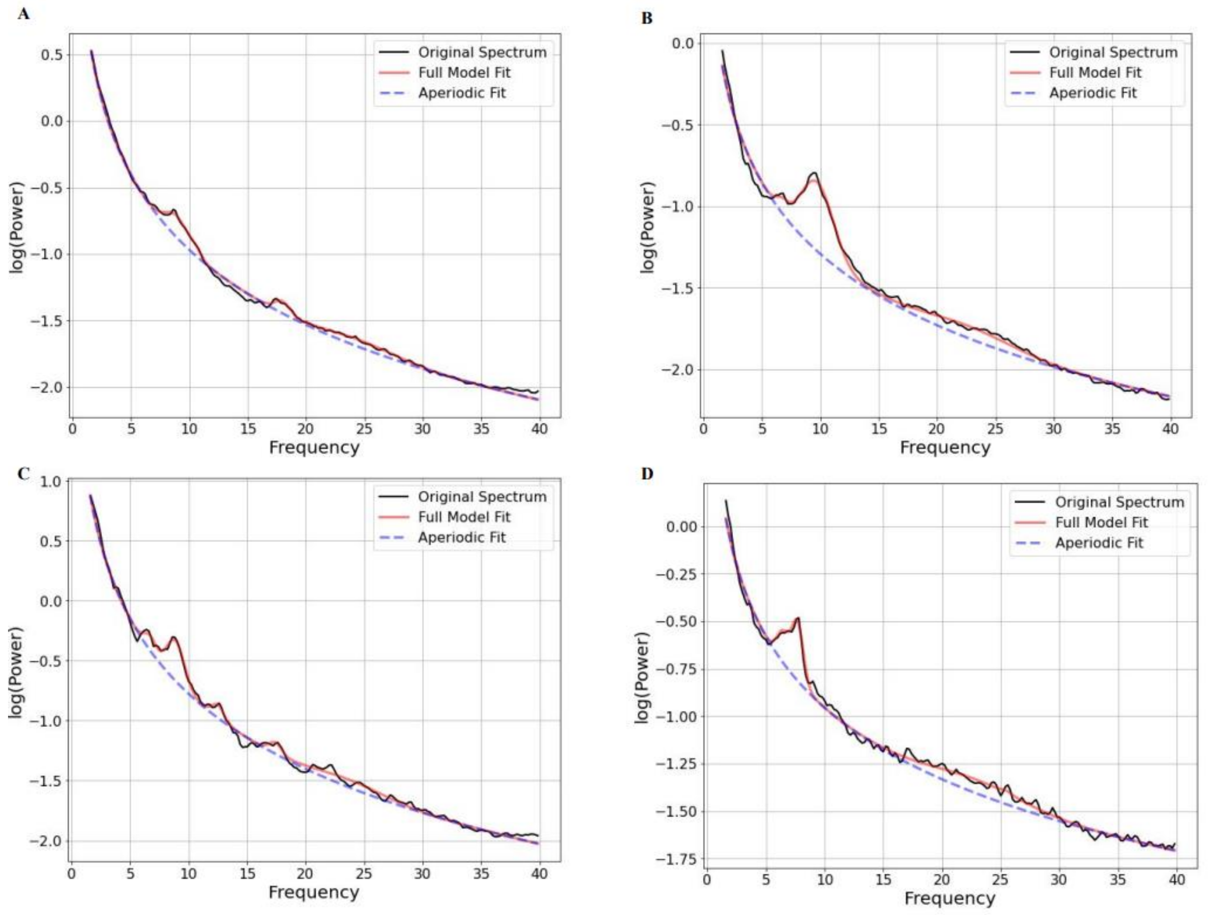

Figure S7| Averaged power spectra plotted for the separate conditions at baseline. A) Individual power spectra averaged over the participants in the learning task who received sham stimulation (n=22). **B)** Individual power spectra averaged over the participants in the learning task who received tRNS (n=16). **C)** Individual power spectra averaged over the participants in the overlearning task who received

sham stimulation (n=21). **D)** Individual power spectra averaged over the participants in the overlearning task who received tRNS (n=16).

Other:

- Why are the other electrodes aside from Fz not analyzed? It would be of interest to see e.g. a spatial specificity, especially in the light of the MRS results.

We would like to thank the reviewer for this point regarding to spatial specificity. To address that we have extracted the baseline aperiodic exponent from electrode T8 (this electrode was chosen since it is not directly related to the FPC, and not under influence of the stimulation electrodes or visual interference). We have completed two analyses: 1) we repeated our original analysis and replaced the baseline aperiodic exponent from electrode Fz with the baseline aperiodic exponent from T8 in our brms model. When we dissected this model with emmeans, we found no significant effect in the learning and stimulation condition which was the case for Fz (lower HPD: -1.06 and upper HPD: 0.66). All other conditions also showed no significant effect. 2) We have repeated our original analysis but in addition, we also included the baseline exponent from T8 as covariate. Such analysis would allow us to not only base our conclusion on a significant model when Fz is included, and a non-significant model when T8 is included, but to examine if the results are specific for Fz. This model with T8 as a covariate still showed a significant effect between learning X tRNS X baseline aperiodic exponent [-2.37, -0.16], while the other conditions were non-significant.
We have added the following in the Results section of the manuscript on line 242: 'To check for spatial specificity, we replaced the exponent from Fz with the exponent calculated over T8, as to the best of our knowledge this electrode has not been linked to mathematical learning. The results show no significant difference for all conditions, specifically for learning X tRNS X baseline aperiodic exponent [-1.06, 0.66]. We also repeated the original model that includes the baseline aperiodic exponent from Fz, but now we controlled for the baseline aperiodic exponent from T8. The three-way interaction was still significant [-2.37, -0.16], further confirming the spatial specificity.'

- Figures have different fonts (e.g. compare Fig. 1 & 2 & 3), font size is too small in some places, pixelated appearance. It would benefit the readibility of this article to improve on these aspects.

We thank the reviewer for this remark, and we have revised our figures accordingly in our manuscript.

- It would be helpful if Fig. 5 showing the structure of the experiment would appear earlier in the text.

In line with the reviewer's suggestion we have placed Figure 5 (now Figure 2) at the end of the Introduction. We also included the following description of this Figure on line 126 of the manuscript: 'To do this, participants completed several multiplication problems by answering in a time-sensitive microphone (see Figure 2A). They were allocated in either the learning or overlearning condition (receiving sham or tRNS for 20 minutes) by means of a variance minimization procedure (see Figure 2B). At the beginning and at the end of the experiment, a resting state (rs)-EEG was measured of eight minutes. The stimulation electrodes were placed over F3 and F4 as determined with the international 10/20 system.'

- Fig. 1 A+B: generally, one would refer to periodic activity to the activity exceeding the 1/f-line, so in that sense the legend is confusing. Also the spectrum looks rotated. I am aware that this is a schematic illustration, but it would also be good to see some spectra from the data, as this would also illustrate data quality.

We would like to thank the reviewer for this good suggestion regarding the legend of Figure 1. We have revised the figure accordingly in the manuscript. Additionally, we have included the averaged power

spectra of the four condition (learning-sham, learning-tRNS, overlearning-sham, overlearning-tRNS) in **Figure S7**.

Reviewer #2:
We would like to thank the reviewer for finding multiple merits in our work, while at the same time raising following concerns that need to be addressed:

1) The essential mechanisms that these results reveal need to be better clarified. The paper describes three different results and need to go a little more profound or global than the results themselves. The authors should discuss these results in contexts of revealing a cue to a novel fundamental mechanism or showing evidence against what is generally believed.

We would like to thank the reviewer for highlighting the need to clarify the essential mechanisms of our results, which was indeed missing. To address this, we have built upon a section in the Discussion of our manuscript about stochastic resonance on line 315. 'So far, it has been assumed that tRNS works by enhancing a signal with a near critical signal-to-noise ratio due to introducing noise in the system, described as the phenomenon of stochastic resonance (McDonnell & Abbott, 2009). This allows the enhancement of otherwise weak neural signals, and therefore, an appropriate amount of noise can increase subthreshold signals. Previous studies related this increased signal-to-noise ratio from tRNS to enhanced learning, perception, and cognitive performance, which are related to stochastic resonance (Antal & Herrmann, 2016; Cappelletti et al., 2013; Fertonani et al., 2011; Herpich et al., 2019; Snowball et al., 2013; Van der Groen & Wenderoth, 2016). In line with this theory, some studies show that participants with poor baseline ability show greater beneficial effect compared to those with strong baseline ability (Evans et al., 2018; Harty & Cohen Kadosh, 2019), as is also the case in our study. However, we should note that the mechanism of stochastic resonance is difficult, if not impossible, to prove in the human brain due to the complexity of biological processes (McDonnell & Abbott, 2009; Mcdonnell & Ward, 2011). That said, a study from Battaglina et al. (2023) showed that noise induced by tRNS produces a stochastic resonance like phenomenon in motion detection. The authors speculate that the added noise acts on the sodium channels in the brain, causing a weak depolarization of the cell membrane of the neurons which increases cortical excitability. However, they also point out the limitation that no electrophysiological signals were measured to record cortical excitability. Our findings suggest a working mechanism of tRNS efficacy related to E/I, which is a tangible and testable mechanism. Whether this mechanism is similar (e.g., both optimal E/I and stochastic resonance are characterized by an inverted-U function; Krause et al., 2013; McDonnell & Abbott, 2009; Van der Groen & Wenderoth, 2016) or orthogonal to the stochastic resonance framework is a question for further research.'

2) The result, which is described as one of the most important findings here, is based on no significant difference in the impact of the level of skill acquisition (learning vs. overlearning) on aperiodic exponents. The no significant difference in the primary analysis was based on p=0.062. Although the further Bayesian analysis is greatly appreciated, these statistical analysis results are not sufficiently strong to draw a significant conclusion as in the paper.

We thank the reviewer for this comment. We would like to clarify that the interaction between task and stimulation ($p=.062$) when predicting the E/I, even if it was significant, does not challenge our claims about the tRNS effect, as it would show that tRNS is able to change the effect of task on E/I. Our discussion of the previous literature in terms of the task effect on E/I are still valid as the main effect of task was not significant ($p=.611$). When excluding tRNS from the model, the effect of task on E/I was not significant ($p=.08$). In addition, we would like to highlight that this interaction, whether significant or not, is not the main outcome of our paper. For example, if the tRNS effect on E/I would have been observed only for the learning and not overlearning, this would have not contradict our main claims and theoretical framework that stimulation influences the E/I during learning. In addition, we ran the Bayesian analysis, as indicated as a strength by the reviewer, because in contrast to frequentist statistics

it allows us to accept H0, rather than only reject H1. These analyses support that there is no evidence for the effect of task or task X stimulation on the exponent. Therefore, we have revised the following on line 205 of the manuscript: ‘Our results, as presented in the Supplementary Information, strengthens the conclusion that tRNS impacted the aperiodic exponent, while we found no evidence of an effect of task.’ However, while there was a trend of the effect of tRNS on task (p=.062), this interaction did not receive support at our Bayesian analysis. The existence of such interaction does not negate our conclusion of no evidence of the effect of task on aperiodic exponent. Further studies could examine whether this is replicated in other cognitive domains.

3) Based on the nonsignificant difference in the level of skill acquisition on EI in the current study and differential effects on MRS-based EI, the authors suggested the natures and roles of impacts of aperiodic portents-based EI and MRS-based EI are different. However, the most significant reason for the differential results are likely to be the differences in experimental conditions between the two studies. Several critical parameters, including the brain regions of measured signals, the extent of measured regions, the type of learning, the degrees of task difficulty, and the timescales of training, are fundamentally different between the two studies. Therefore, discussing the possible contribution of differential impacts of aperiodic portent-based EI and MRS-based EI is premature.

We apologize if we gave the impression that the MRS-based E/I and the EEG-based EEG were measured during a task or intervention and we would like to clarify that this is not the case. These are baseline measures that took place before the intervention/task manipulation. Therefore, the differences could not be due to differential experimental conditions, task difficulty, type of learning, and the timescales of the training. This information was missing in the previous version and we have clarified this by adding the following part in the discussion of the manuscript: ‘during rest before the intervention took place’ on line 398. In addition, the effects are also not due to differences in the measured brain regions. As mentioned in **Figure S4**, the GABA and glutamate levels are measured by MRS over the left IPS and the left MFG. These areas correspond to the EEG electrodes P3 and F3.

4) Why the authors applied tRNS to DLPFC, not other regions, needs to be clarified. To discuss the general nature and impact of tRNS on aperiodic exponents, it is necessary to conduct experiments in which tRNS is applied to several different important brain regions or to give a strong rationalization as to why it was applied only to DLPFC.

We would like to thank the reviewer for recognizing the lack of clarity regarding the chosen brain areas that are targeted with tRNS (i.e., the left and right DLPFC). Our rational for choosing to stimulate at F3 and F4, is that the left and right DLPFC play an important role at the beginning of learning before expertise is gained. This is based on multiple neuroimaging studies in the field of mathematical learning and cognition (for reviews see Zamarian et al. 2009, Neuroscience and Biobehavioral Reviews, https://doi.org:10.1016/j.neubiorev.2009.03.005; Zamarian & Delazer, 2015, in The Oxford Handbook of Numerical Cognition (eds R. Cohen Kadosh & A. Dowker, Oxford University Press). These findings are in line with non-mathematical studies in the field of cognitive learning (Chein & Schneider, 2012, Current Directions in Psychological Science). In previous tRNS studies we have already documented the effect of tRNS over DlPFC, and not on other regions (posterior parietal cortex) (Snowball et al., 2013, supplementary information; and replicated in Zacharopoulos et al., submitted).

These neuroimaging and neurostimulation studies, as well as the theoretical framework by Chein and Schneider (2012) served the basis for the application of tRNS only to DLPFC. To clarify this in the manuscript, we have added the following in the Methods on line 514: ‘Based on previous neuroimaging and tRNS experiments, we targeted these frontal areas due to its involvement in the early phases of mathematical learning, rather than other brain regions such as the parietal cortex (Snowball et al., 2013; Zacharopoulos et al., submitted; Zamarian et al., 2009; Zamarian & Delazer, 2015). These findings are in line with non-mathematical studies in the field of cognitive learning (Chein & Schneider, 2012).’

5) learning/overlearning is one of many different types of the level of skill acquisition. It is premature to draw a general conclusion about the level of skill acquisition from the results of an experiment in which only learning/overlearning was manipulated.

We agree with the reviewer and to avoid confusion we removed this phrasing from the manuscript.
