# Supplementary material for: Human neuronal excitation/inhibition balance explains and predicts neurostimulation induced learning benefits
Source: PLoS Biol. 2023 Aug 31;21(8):e3002193. doi: 10.1371/journal.pbio.3002193 (PMC10470965; doi:10.1371/journal.pbio.3002193)
Supplement: Attachment — Submitted filename: Response to Reviewers2.pdf [file pbio.3002193.s004.pdf]

Tracking Number [PBIOLOGY-D-22-02703R1]

Dear Editors,

We would like to express our gratitude for your response to our resubmission of the manuscript with the revised title: 'Human Neuronal Excitation/Inhibition Balance Explains and Predicts Neurostimulation Induced Learning Benefits'. We appreciate the diligent efforts of the editorial board and we would like to thank the new reviewer 3 for evaluating our work and providing constructive feedback. We have addressed the comments made by reviewer 3 and we describe below how this has been done on a point-by-point basis. Revisions that were made in the manuscript are non-indented and blue in the rebuttal letter and marked as track changes in the revised manuscript.

Kind regards,

Roi Cohen Kadosh & Nienke van Bueren in the name of the authors

Reviewer #3:

1) …The authors have done a fine job at addressing point (2), though I would also like to see some EEG topoplots for the FOOOF parameters, instead of also just comparing Fz to T8.

We would like to thank the reviewer for the suggestion to include topoplots for the FOOOF aperiodic exponent. In response to this suggestion, we have included the topoplots of the aperiodic exponent showing the change from baseline to post in Figure 4 of the manuscript. To provide a comprehensive analysis, we have extracted the topoplots for both the sham and tRNS group, which complement Figure 4. These plots illustrate the increase in excitation/inhibition (E/I), as indicated by a decrease in the exponent, induced by tRNS over electrode Fz compared to sham. We refer to these plots on line 181 of the manuscript: 'The topographies show a clear decrease in the aperiodic exponent after tRNS opposed to sham stimulation for the anterior electrodes, indicating an increased E/I (see **Figure 4B**)'. And also on line 248: 'To check for spatial specificity, we replaced the exponent from Fz with the exponent calculated over T8, as to the best of our knowledge this electrode has not been linked to mathematical learning (also see Figure 4B for topographical comparisons).'

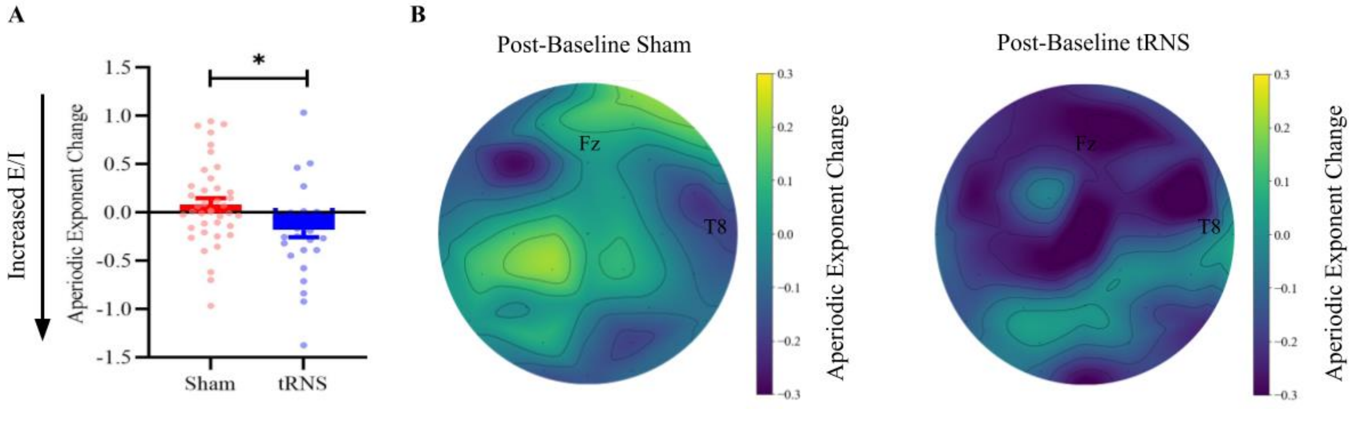

Figure 4| Changes in aperiodic exponent for active and sham tRNS and their associated topographies. A) Participants who received active tRNS showed an increase in E/I as indicated by the mean (±S.E.M.) decreased aperiodic exponent (change: post-baseline exponent in μV^2Hz^{-1}). Participants who received sham tRNS showed a mean (±S.E.M.) decrease in E/I as indicated by an increased exponent. **p*<.05. **B)** Topoplot illustrates the change in the aperiodic exponent for the sham (left) and active tRNS (left and right respectively). For electrode Fz, a slight increase in the aperiodic exponent is

observed as indicated with a lighter color for sham tRNS. For active tRNS there is a clear decrease in the aperiodic exponent after tRNS, as indicated with a darker color for electrode Fz and anterior electrodes. This observation supports the notion of increased excitation/inhibition (E/I) following active compared to sham tRNS.

*2) Regarding the more serious point (1), the authors are correct that there are many rapidly emerging, converging points of evidence that lend support to the theoretical validity. Some of these concerns can be mitigated by softening the language a bit, to emphasize that this is a *putative* marker for EI. The authors do this once now, in the Introduction, but adding that caveat to the Abstract and in the Discussion would be better. In brief: this is an interesting paper, and the authors have done a fine job with the revision.*

We agree with the reviewer and we have softened the language in the abstract (line 25) and in the discussion (line 405) of the manuscript.
